# Reliability of BRAF mutation detection using plasma sample: A protocol for systematic review and meta-analysis: Erratum

**DOI:** 10.1097/MD.0000000000028577

**Published:** 2022-01-14

In the article, “Reliability of BRAF mutation detection using plasma sample: A protocol for systematic review and meta-analysis”^1^, which appears in Volume 100, Issue 51 of *Medicine*, “a protocol” was incorrectly included in the title. The title has been corrected to “Reliability of BRAF mutation detection using plasma sample: A systematic review and meta-analysis.”
